# Incidental dural tears associated with worse clinical outcomes in patients operated for lumbar spinal stenosis

**DOI:** 10.1007/s00701-022-05421-5

**Published:** 2022-11-18

**Authors:** Ole Kristian Alhaug, Filip Dolatowski, Ivar Austevoll, Sverre Mjønes, Greger Lønne

**Affiliations:** 1 Innlandet Hospital Trust, Brumunddal, Norway; 2 Akershus University Hospital, Nordbyhagen, Norway; 3 Norwegian University of Science and Technology, Trondheim, Norway; 4 Division of Orthopaedic Surgery, Oslo University Hospital, Oslo, Norway; 5 Helse Bergen HF, Bergen, Norway

**Keywords:** Dural tear, Failure, Worsening, Spine registry, Lumbar spinal stenosis

## Abstract

**Study design:**

Retrospective cohort study.

**Objective:**

Incidental dural (ID) tear is a common complication of spine surgery with a prevalence of 4–10%. The association between ID and clinical outcome is uncertain. Former studies found only minor differences in Oswestry Disability Index (ODI). We aimed to examine the association of ID with treatment failure after surgery for lumbar spinal stenosis (LSS).

**Methods:**

Between 2007 and 2017, 11,873 LSS patients reported to the national Norwegian spine registry (NORspine), and 8,919 (75.1%) completed the 12-month follow-up. We used multivariate logistic regression to study the association between ID and failure after surgery, defined as no effect or any degrees of worsening; we also compared mean ODI between those who suffered a perioperative ID and those who did not.

**Results:**

The mean (95% CI) age was 66.6 (66.4–66.9) years, and 52% were females. The mean (95% CI) preoperative ODI score (95% CI) was 39.8 (39.4–40.1); all patients were operated on with decompression, and 1125 (12.6%) had an additional fusion procedure. The prevalence of ID was 4.9% (439/8919), and the prevalence of failure was 20.6% (1829/8919). Unadjusted odds ratio (OR) (95% CI) for failure for ID was 1.51 (1.22–1.88); *p* < 0.001, adjusted OR (95% CI) was 1.44 (1.11–1.86); *p* = 0.002. Mean postoperative ODI 12 months after surgery was 27.9 for ID vs. 23.6 for no ID.

**Conclusion:**

We demonstrated a significant association between ID and increased odds for patient-reported failure 12 months after surgery. However, the magnitude of the detrimental effect of ID on the clinical outcome was small.

## Introduction

Incidental durotomy (ID) is the most common perioperative complication of spinal surgery. A dural tear usually leads to cerebrospinal fluid leakage (CSF), and nerve filaments may erupt and become damaged. The prevalence of ID in spinal stenosis surgery ranges from 4 to 10% [7, 8, 14, 24, 29, 31], and ID is associated with an increased risk for neurological deficit, revision surgery, longer hospital stay, and increased treatment costs [7, 14, 30]. However, the effect of ID on clinical outcomes is debated, and previously published data is somewhat conflicting. A systematic review found minor differences in Oswestry Disability Index (ODI) scores between patients with and without ID [5]. Interpreting differences in patient-reported outcome measures (PROMs) between groups may be challenging, and few studies have used dichotomous endpoints. However, two register studies reported dichotomous patient satisfaction and found that patients who suffered an ID were less satisfied despite minor differences in PROM scores [14, 29].

Factors such as previous surgery may confound the effect of ID on outcome measures by affecting both the risk for ID and the clinical outcome. However, confounding factors are not always adjusted for in studies of ID.

The aim of this retrospective observational study on prospectively collected national spine register data was to assess the association between ID and failure and worsening after surgery for lumbar spinal stenosis (LSS).

## Methods

This retrospective study on prospectively collected data included adult patients operated on for lumbar spinal stenosis (LSS) between 2007 and 2017 in Norway. Patients were included in the Norwegian Registry of Spine Surgery (NORspine), a comprehensive national registry for quality control and research. The coverage is 70%, and the 12-month loss to follow-up is 26% [27]. The registration includes informed consent and patient and surgeon-reported data.

Before surgery, patients report symptoms of their spinal disease by standardized PROMs and general health status, quality of life, and socioeconomic status. Immediately after surgery, surgeons report details concerning the spinal diagnosis, relevant comorbidities, and surgical details, including any perioperative complications. Three months after surgery, patients report directly to NORspine by regular mail on the effect of surgery by common PROMs. The PROMs are the Norwegian translation of the Oswestry Disability Index (ODI), a pain-related disability score ranging from 0 (no impairment) to 100 (bedbound) [10, 11, 13], Numerical Rating Scale (NRS) for back and leg pain (ranging from 0 = no pain to 10 = worst pain imaginable) [19], and global perceived effect (GPE) scale — a seven-step transition scale (1 = completely recovered, 2 = much improved, 3 = somewhat improved, 4 = unchanged, 5 = somewhat worse, 6 = much worse, 7 = worse than ever) [17]. Patients also report any postoperative complications at 3 months. Finally, 12 months after surgery, patients repeatedly grade the effect of surgery on their symptoms by the PROMs mentioned above.

### Primary outcome

Failure was defined as patients who perceived themselves as unchanged or any degree of worse after surgery (GPE 4–7). Worsening was defined as patients who perceived themselves as “much worse” or “worse than ever” after surgery (GPE 6–7).

### Secondary outcomes

Failure and worsening were also defined by cutoffs for ODI final score, ODI absolute difference (postoperative minus preoperative), and ODI percentage change according to previously published definitions [2]. The ODI cutoffs for failure used in this study were ODI final score > 31, ODI absolute improvement < 8 points, and ODI percentage change < 20%. The corresponding cutoffs used to define worsening were ODI final score > 39, ODI absolute improvement < 4 points, and ODI percentage change < 9% [2]. We also used mean ODI final score to assess the impact of ID on patient-reported outcomes after surgery and mean NRS leg as an indirect measure of the association between ID and neurologic symptoms after surgery. Finally, we registered the length of hospital stay and patient-reported postoperative complications.

## Statistics

### Descriptive statistics

The study population was described using means (95% CIs) for continuous data and numbers and proportions for categorical data.

### Primary outcome

To estimate the association between ID and clinical outcomes, we used multiple logistic regression with failure and worsening (defined by GPE) as dependent variables, ID (yes/no), and potential confounders as independent variables. Based on previously published data, we adjusted the primary analysis by the following potential confounders: age, gender, BMI, smoking, ASA (dichotomized as grades 1 and 2 vs. grades 3, 4, and 5), preoperative PROMs, duration of leg pain before surgery, previous surgery (at the same lumbar level), multilevel surgery, and fusion (in addition to decompression) [1, 4, 21, 22]. The potential confounders were decided a priori and not by statistical testing. We provide unadjusted and adjusted estimates for odds ratios (OR) with 95% CIs.

### Secondary outcomes

To examine the secondary outcomes, we repeated the regression analysis using the different dichotomous outcomes (ODI final score, ODI absolute change, and ODI percentage change). To quantify the association between ID and the mean ODI final score and NRS leg pain score, we used multiple linear regression with ODI final score and NRS leg pain as dependent variables, adjusting for the aforementioned possible confounders. We also analyzed the association between ID and length of hospital stay and patient-reported postoperative complications, using multiple linear regression and multiple logistic regression, adjusting for possible confounders. We did not impute any missing data.

We used SPSS, version 26 (IBM Corp., Armonk, NY, USA) for the statistical analyses.

### Ethics

Participation in NORspine is voluntary and presumes written consent. The study was also approved by The Norwegian Regional Committee for medical and health research ethics (2017/2157). The study was conducted in accordance with the Helsinki declaration, and we have reported the results in line with the STROBE guidelines.

## Results

We identified 11,873 NORspine patients operated for LSS during 10 years (January 2007 to April 2017). A total of 8863 (74.6%) patients completed the 3-month follow-up, and 8919 (75.1%) responded 12 months after surgery. The mean (95% CI) age of the study population was 66.6 (66.4–66.9) years, and 4384 (52%) were females. The mean (95% CI) preoperative ODI was 39.8 (39.4–40.1). All patients were operated on with decompression, and 1125 (12.6%) had an additional fusion procedure. Patient characteristics and baseline data for PROMS are shown in Table 1. Table 1 also shows that 1829 (20.6%) reported a GPE score corresponding to failure, and 521 (5.9%) reported a GPE corresponding to worsening after surgery. Surgeons reported incidental durotomy in 439 cases (4.9%).

**Table 1:** Patient characteristics and clinical outcome for 8908 Norwegian patients with surgically treated lumbar spinal stenosis and completed the 12-month follow-up, broken down by no incidental dural tear and incidental dural tear

	No incidental dural tear (*n* = 8427) Mean (95% CI) or *n* (%)	Incidental dural tear (*n* = 436) Mean (95% CI) or *n* (%)
Age	66.5 (66.3–66.7)	69.2 (68.2–70.1)
Female gender	4384 (51.7%)	260 (59.2%)
Civil status, single	2164 (25.5%)	118 (26.9%)
Norwegian as 1st language	8201 (96.7%)	421 (95.9%)
ASA (grade 3 to 5)*	1732 (20.7%)	108 (24.7%)
Body mass index	27.5 (27.4–27.6)	27.8 (27.4–28.4)
Smoking	1629 (19.4%)	68 (15.6%)
University or college education	2506 (29.6%)	120 (27.3%)
Receives disability benefit	1198 (14.1%)	67 (15.3%)
Previous spinal surgery (same level)	1133 (13.5%)	100 (23.1%)
Leg pain > 12 months before surgery	5023 (64.2%)	261 (65.3%)
Additional fusion (any type)	1050 (12.4%)	75 (17.1%)
Preoperative ODI**	39.6 (39.3–40.0)	42.1 (40.6–43.6)
Preoperative NRS leg pain***	6.56 (6.51–6.61)	6.68 (6.45–6.90)
Preoperative NRS back pain***	6.49 (6.44–6.54)	6.70 (6.49–6.92)
Preoperative EQ-5D****	0.377 (0.370–0.384)	0.355 (0.323–0.388)
More than one level operated	3024 (36.0%)	231 (53.2%)
Failure 12 months (missing 56)*****	1708 (20.3%)	121 (27.8%)
Worsening 12 months (missing 56)******	480 (5.7%)	41 (9.4%)
ODI final score	23.6 (23.3–24.0)	27.9 (26.2–29.6)
Length of stay (days)	3.31 (3.24–3.39)	5.68 (5.17–6.20)
Postoperative complications, any (*n* = 8882)	1154 (13.7%)	105 (23.3%)

^*^American Society of Anesthesiologists classification (1–5), increasing for worse health

^**^Oswestry Disability Index (0–100), increasing for increasing disability

^***^Numeric Rating Scale (0–10), increasing for increasing pain

^****^EuroQol’s quality of life (− 0.60 to 1.00), increasing for better quality of life

^*****^Defined as global perceived effect (GPE) 4 + 5 + 6 + 7 (unchanged or any degree of worsening) at 12 months

^******^Defined as global perceived effect (GPE) 6 + 7 (“much worse” or “worse than ever”) at 12 months

A total of 1708 (20.3%) patients without ID perceived treatment as failure, compared to 121 (27.8%) patients with ID. A total of 480 (5.7%) patients without ID reported worsening, compared to 41 (9.4%) patients with ID. Compared to patients with no perioperative ID, patients who suffered an ID more often reported failure (adjusted OR (95% CI) 1.45 (1.12–1.87); *p* = 0.005) and worsening (adjusted OR (95% CI) 1.50 (1.01–2.23); *p* = 0.045 (Table 2). Patients who suffered an ID during spinal surgery more often reported failure and worsening assessed by other outcome measures (Table 3).

**Table 2:** Multiple logistic regression using “failure”* and “worsening”** at 12-month follow-up as the dependent variable and dural tear and potential confounders as covariates

Variables	Failure		Worsening	
	OR (95% CI)	*p*-value	OR (95% CI)	*p*-value
Dural tear	1.45 (1.12–1.87)	0.005	1.50 (1.01–2.23)	0.045
Age	1.00 (1.00–1.01)	0.312	1.00 (0.99 –1.01)	0.547
Gender (female)	0.88 (0.78–1.00)	0.043	0.97 (0.78–1.20)	0.769
Body mass index (cont)	1.01 (1.00–1.03)	0.028	1.00 (0.97–1.02)	0.659
Smoking	1.41 (1.22–1.64)	0.000	1.53 (0.93–1.54)	0.001
ASA (3 + 4 + 5)***	1.13 ( 0.97–1.31)	0.125	1.20 ( 0.94–1.54)	0.153
Preoperative ODI (cont)****	1.01 (1.01–1.02)	0.000	1.03 (1.02–1.04)	0.000
Preoperative NRS leg pain*****	0.93 (0.89–0.96)	0.000	0.96 (0.90–1.02)	0.202
Preoperative NRS back pain*****	1.10 (1.05–1.14)	0.000	1.15 (1.07–1.24)	0.000
Duration leg pain > 12 months	1.63 (1.43–1.87)	0.000	1.54 (1.22–1.94)	0.000
Former surgery at same level	1.92 (1.64–2.26)	0.000	1.79 (1.38–2.31)	0.000
More than one level operated	0.90 ( 0.79–1.02)	0.092	0.93 ( 0.74–1.15)	0.490
Additional fusion, any type	0.62 (0.51–0.75)	0.000	0.61 (0.43–0.85)	0.003

^*^Defined as global perceived effect (GPE) 4–7 (unchanged or any degree of worsening) at 12 months

^**^Defined as global perceived effect (GPE) 6 + 7 (“much worse” or “worse than ever”) at 12 months

^***^American Society of Anesthesiologists classification (1–5) (grades 3 to 5)

^****^Oswestry Disability Index (0–100), increasing for increasing disability

**Table 3:** Associations between dural tear and various dichotomous clinical outcomes in repeated multiple logistic regression analyses, adjusted for the same variables as in the main model (Table 2), displaying only the main results (effects of dural tear)

Clinical outcome, dichotomized		OR (95% CI)
GPE	Failure (GPE 4–7)*	1.45 (1.12–1.87)
	Worsening (GPE 6 + 7)**	1.50 (1.01–2.23)
ODI final score (12 months)	failure (ODI > 31)***	1.29 (1.00–1.67)
	worsening (ODI > 39)***	1.43 (1.08–1.88)
ODI absolute difference	Failure (< 8 points improvement)***	1.15 (0.91–1.47)
	Worsening (< 4 points improvement)***	1.28 (1.00–1.65)
ODI percentage difference	Failure (< 20% improvement)***	1.17 (0.93–1.49)
	Worsening (< 9% improvement)***	1.25 (0.96–1.61)

^*^Global perceived effect scale 4–7 (unchanged or any degree of worsening)

^**^Global perceived effect scale 6 + 7 (“much worse” or “worse than ever”)

^***^Oswestry Disability Index (ODI) cutoffs selected from previous published study (15)

Patients who suffered an ID during surgery reported lower ODI 12 months after surgery than those who did not suffer an ID (23.6 (23.3–24.0)) vs. 27.9 (26.2–29.6). This difference remained significant after adjusting for possible confounders (beta (95% CI) = 2.29 (0.58–4.00); *p* = 0.009) (Tables 1 and 4; Appendix Table 7). Also, patients who suffered an ID reported more leg pain after surgery compared to patients without ID: mean NRS leg pain was 4.2 (3.9–4.5) vs. 3.5 (3.5–3.6); this difference remained significant after adjusting for confounders (beta (95% CI) of 0.6 (0.3–0.9); *p* < 0.001) (Table 4; Appendix Table 8). Patients with ID had longer hospital stays than patients without ID (mean (95% CI) 5.7(5.2–6.2) vs. 3.3 (3.2–3.4) (Table 1). This difference remained significant after adjusting for confounders (beta (95% CI) 1.58 (1.25–1.92) days; *p* < 0.001 (Table 4; Appendix Table 9).

**Table 4:** Linear regression analyses displaying the effect of dural tear on “length of stay,” “ODI final score,” and “NRS leg pain,” adjusted for the same potential confounders as Table 2, displaying only the main results (see Appendix Tables 7, 8, and 9 for complete results)

Variable	Beta	95% CI	*p*-value
Length of stay*	1.58	1.25–1.92	0.000
ODI final score**	2.29	0.58–4.00	0.009
NRS leg pain***	0.6	(0.3–0.9)	0.000

^*^Length of stay, days

^**^Oswestry Disability Index (0–100), increasing for increasing disability

^***^Numeric Rating Scale (0–10), increasing for increasing leg pain

Among respondents at a 3-month follow-up, 1259 (14.2%) patients reported any postoperative complication. The corresponding numbers for patients without ID were 1154 (13.7%), and for patients with ID, 105 (23.3%) (Table 1). The multiple logistic regression showed that patients with ID had increased odds of urinary tract infection (UTI) after surgery (OR (95% CI) 2.42 (1.53–2.73); *p* < 0.001). However, the odds for other postoperative complications were not increased for patients with ID (Table 5).

**Table 5:** Main results from multiple logistic regression analyses showing odds ratios for the association between incidental dural tear and postoperative complications, *n* = 8882 (patients answered at 3-month follow-up), adjusted for the same potential confounders as in Table 2

	OR	95% CI	*p-*value
Postoperative complications, in total	1.42	0.96–2.10	0.078
Infection, superficial	0.45	0.14–1.47	0.187
Infection, deep	0.62	0.78–4.91	0.650
Deep venous thrombosis	0.00	0.00–	0.995
Pulmonary embolism	0.00	0.00–	0.996
Pneumonia	0.62	0.15–2.69	0.527
Urinary tract infection	2.42	1.53–2.73	0.000
Micturition problems	1.46	0.78–2.73	0.232

All postoperative complications are patient reported only; there is no medical confirmation of the postoperative complications in the NORspine registry

We found that the following covariates affected the odds for failure (GPE 4–7) and worsening (GPE 6–7) after surgery for lumbar stenosis: gender, BMI, preoperative PROMs, preoperative duration of leg pain, former surgery at the same lumbar level, a fusion procedure, and smoking (Table 2). Table 6 displays possible risk factors for ID: age, gender (female), former surgery, and multilevel operations increased the odds of ID.

**Table 6:** Multiple logistic regression displaying the effect of the potential confounders on the odds for dural tear

Variables	OR (95% CI)	*p*-value
Age	1.02 (1.01–1.04)	0.000
Gender (female)	1.36 (1.08–1.72)	0.010
Body mass index (cont)	1.02 (1.00–1.05)	0.075
Smoking	0.93 (0.68–1.27)	0.652
ASA (3 + 4 + 5)*	1.08 ( 0.82–1.43)	0.567
Preoperative ODI (cont)**	1.01 (1.00–1.02)	0.159
Preoperative NRS leg pain***	0.98 (0.92–1.04)	0.425
Preoperative NRS back pain***	1.02(0.95–1.09)	0.616
Duration leg pain > 12 months	0.92 (0.73–1.16)	0.458
Former surgery at same level	1.94 (1.48–2.54)	0.000
More than one level operated	1.84 (1.47–2.31)	0.000
Additional fusion	1.12 (0.82–1.53)	0.476

^*^American Society of Anesthesiologists (ASA) classification (1–5) (grade 3 to 5)

^**^Oswestry Disability Index (0–100), increasing for increasing disability

^***^Numeric Rating Scale (0–10), increasing for increasing pain

## Discussion

### Main findings

This retrospective study on prospectively collected data from a nationwide register of nearly nine thousand patients operated for lumbar spinal stenosis demonstrated a significant association between incidental dural tear (ID) and increased odds for patient-reported failure and worsening 12 months after surgery. However, the magnitude of the detrimental effect of ID on the outcome of spinal surgery was small. The main finding was supported by analyses of secondary endpoints, including using different PROM derivates that defined treatment failure and worsening after surgical treatment of lumbar spinal stenosis.

A dural tear may vary from a small partial puncture of the dura, with an intact arachnoidea, to a large defect with gross leakage of cerebrospinal fluid (CSF) and damaged nerve filaments. Also, the repair of the dural tear may vary from a waterproof dural suture to an incomplete repair with continuous leakage of CSF. A surgical repair of a dural tear may also potentially traumatize nerve filaments. Clinical outcomes are expected to differ between the extremes mentioned above of dural tear and repair; however, NORspine data on IDs do not differentiate between different grades of ID or various surgical repairs. We cannot rule out that the increased risk for failure and worsening associated with ID may be attributed to the most severe IDs and incomplete repairs.

Several studies have reported minor detrimental effects of ID on PROMs, with uncertain clinical importance effect sizes [5, 8, 16, 25, 28, 29]. One large register study from Sweden [29] found only a minor increase in postoperative ODI associated with ID but still a significantly inferior patient satisfaction (a categorical variable) associated with ID. Similarly, a Tango spine register study found no effect of ID on core outcome measure index (COMI) scores but a trend toward lesser patient-reported satisfaction among patients with an ID [14]. Small to moderate between-groups differences in proportions of failures are often associated with only minor differences in absolute PROM scores. Our partly conflicting results between dichotomous and continuous outcome measures reflect the small effect ID has on ODI scores and are in line with the studies mentioned above. This illustrates the importance of using categorized outcomes in addition to mean PROM values. Categorized outcomes are also emphasized in a review article on clinical important change [18].

### Secondary outcomes

Patients who suffered a dural tear needed longer hospitalization, a finding consistent with previously published data [7, 8, 14]. Current practice is to advise (one or) a few days of bed rest after IDs with CSF leakage, especially when patients report spinal headaches. Bedrest after IDs may be a primary reason for prolonged hospitalization. Observation, further diagnostic tests, and reoperations may explain the prolonged stay in patients with IDs.

In this (cohort) study, IDs were associated with increased odds of postoperative urinary tract infection, however not for other patient-reported postoperative complications. Previous studies have shown that IDs may be associated with increased risk for other postoperative complications such as wound infections, neural deficits, postoperative delirium, and perioperative blood loss [7, 8, 30]. NORspine records patient-recorded complications at 3 months after surgery. Patients may define complications differently from health care personnel. The NORspine design and complication recording probably explain the difference between our findings and previously published data on the impact of ID on postoperative complications.

This study was specifically designed to assess the effect of ID on the odds of failure and worsening after surgical treatment of lumbar stenosis. However, as presented in Table 2, other baseline variables such as preoperative ODI, duration of leg pain, and previous lumbar surgery also affected the odds of failure and worsening — in line with previous studies [1, 22]. However, our study was not designed to numerically assess the predictors for failure and worsening. Our finding of increased odds for ID with increasing age and previous surgery is also in line with an earlier Tango register study [14].

### Limitations

NORspine data do not differentiate between different grades of ID or different managements of ID. This may obscure essential factors that may affect the impact of ID on clinical outcome. Furthermore, surgeons are likely to underreport dural tears. In a former validation study of NORspine data against electronic patient records, the authors demonstrated a sensitivity of 40% for the actual reporting of perioperative complications [3]. Underreporting of complications has been shown in other registers [20, 23] and could have affected conclusions regarding the impact of ID on clinical outcomes in other studies. Although the NORspine frequency of IDs (4.9%) is comparable to some reports [7, 8, 14, 24, 29, 31], it is inferior to the prevalence of IDs (9–11%) in two large RCTs on similar patient groups [6, 12]. The underreporting of ID may contribute to underestimating the effect of ID on clinical outcome.

ID may lead to neurological damage. NORspine does not record postoperative neurological sequelae or liquorrhea. However, we used NRS leg pain as a surrogate variable to assess neurological sequelae.

Furthermore, *postoperative* complications registered in NORspine are only reported by patients 3 months after surgery, and this register design may miss some postoperative complications. Postoperative complications may have been treated at different medical centers or in primary care, and complete complication data are unavailable in NORspine.

At 12 months after surgery, NORspine demonstrates a loss to follow-up of 26% [27]. According to former validation studies of medical registers, loss to follow-up does not systematically bias clinical outcomes [9, 15, 26]. One of these studies [26] specifically examined the impact of loss to follow-up in the NORspine registry.

Our choice of GPE as the primary outcome can be discussed. GPE may be susceptible to recall bias. However, GPE has shown excellent test re-test reliability in studies of musculoskeletal disorders [17].

## Conclusion

This prospective nationwide spine register study found that incidental dural tear was associated with increased odds of patient-reported failure and worsening after surgery for lumbar spinal stenosis. The detrimental association to the clinical outcome was small and could be attributed to the most severe dural tears. Incidental dural tear was also associated with increased length of stay.

## Appendix

See Tables 7

**Table 7:** Quantifying the association between dural tear and final ODI score using multiple linear regression, “ODI 12 months” as the dependent variable, and dural tear and potential confounders as independent variables

Variables	*B* (95% CI)	*p*-value
Dural tear	2.29 (0.58 to 4.00)	0.009
Age	0.05(0.01 to 0.08)	0.019
Gender (female)	1.05 (0.29 to 1.81)	0.007
Body mass index (cont)	0.28 (0.19 to 0.36)	0.000
Smoking	3.56 (2.61 to 4.51)	0.090
ASA (3 + 4 + 5)*	2.80 (1.83 to 3.77)	0.000
Preoperative ODI (cont)**	0.50 (0.47 to 0.53)	0.000
Preoperative NRS leg pain***	− 0.80 (− 1.02 to − 0.59)	0.000
Preoperative NRS back pain***	0.89 (0.66 to 1.11)	0.000
Duration leg pain > 12 months	4.98 (4.21 to 5.74)	0.000
Former surgery at same level	6.40 (5.33 to 7.46)	0.000
More than one level operated	− 0.04 (− 0.82 to 0.73)	0.917
Additional fusion	− 2.52 (− 3.65 to − 1.39)	0.000

^*^American Society of Anesthesiologists (ASA) classification (1–5) (grades 3 to 5)

^**^Oswestry Disability Index (0–100), increasing for increasing disability

^***^Numeric Rating Scale (0–10), increasing for increasing pain

, 8

**Table 8:** Quantifying the association between dural tear and NRS leg pain score using multiple linear regression, “NRS leg pain” as the dependent variable, and dural tear and potential confounders as independent variables

Variables	*B* (95% CI)	*p*-value
Dural tear	0.57 (0.26 to 0.89)	0.000
Age	0.01(0.0 to 0.02)	0.023
Gender (female)	0.62 (− 0.8 to 0.20)	0.377
Body mass index (cont)	0.03 (0.02 to 0.05)	0.000
Smoking	0.44 0.28 to 0.62)	0.000
ASA (3 + 4 + 5)*	0.28 (0.11 to 0.46)	0.000
Preoperative ODI (cont)**	0.03 (0.02 to 0.03)	0.000
Preoperative NRS leg pain***	0.11 (0.08 to 0.15)	0.000
Preoperative NRS back pain***	0.11 (0.07 to 0.15)	0.000
Duration leg pain > 12 months	0.79 (0.65 to 0.93)	0.000
Former surgery at same level	0.94 (0.75 to 1.13)	0.000
More than one level operated	− 0.13 (− 0.27 to 0.01)	0.078
Additional fusion	− 0.62 (− 0.83 to − 0.42)	0.000

^*^American Society of Anesthesiologists (ASA) classification (1–5) (grade 3 to 5)

^**^Oswestry Disability Index (0–100), increasing for increasing disability

^***^Numeric Rating Scale (0–10), increasing for increasing pain

, and 9

**Table 9:** Quantifying the association between dural tear and length of stay, using multiple linear regression, “length of stay” as the dependent variable, and dural tear and potential confounders as independent variables

Variables	*B* (95% CI)	*p*-value
Dural tear	1.58 (1.25 to 1.92)	0.000
Age	0.03(0.02 to 0.04)	0.000
Gender (female)	0.50 (0.35 to 0.64)	0.000
Body mass index (cont)	0.02 (0.00 to 0.04)	0.000
Smoking	− 0.16 (− 0.35 to 0.03)	0.090
ASA (3 + 4 + 5)*	0.35 (0.17 to 0.54)	0.000
Preoperative ODI (cont)**	0.03 (0.02 to 0.02)	0.000
Preoperative NRS leg pain***	− 0.07 (− 0.12 to − 0.3)	0.001
Preoperative NRS back pain***	0 − 01 (− 0.04 to 0.05)	0.691
Duration leg pain > 12 months	0.25 (0.10 to 0.40)	0.001
Former surgery at same level	0.22 (0.01 to 0.43)	0.042
More than one level operated	0.81 (0.65 to 0.96)	0.000
Additional fusion	3.54 (3.31 to 3.76)	0.000

^*^American Society of Anesthesiologists (ASA) classification (1–5) (grade 3 to 5)

^**^Oswestry Disability Index (0–100), increasing for increasing disability

^***^Numeric Rating Scale (0–10), increasing for increasing pain

.
